# Genome‐wide population structure and admixture analysis reveals weak differentiation among Ugandan goat breeds

**DOI:** 10.1111/age.12631

**Published:** 2018-01-17

**Authors:** R. B. Onzima, M. R. Upadhyay, R. Mukiibi, E. Kanis, M. A. M. Groenen, R. P. M. A. Crooijmans

**Affiliations:** ^1^ Wageningen University & Research Animal Breeding and Genomics P O Box 338, 6700AH Wageningen The Netherlands; ^2^ National Agricultural Research Organization (NARO) P O Box 295 Entebbe Uganda; ^3^ Department of Animal Breeding and Genetics Swedish University of Agricultural Sciences Uppsala Sweden; ^4^ Department of Agriculture, Food and Nutritional Sciences (AFNS) Faculty of Agriculture, Life and Environmental Sciences University of Alberta 1416 College Plaza Edmonton T6G 2C8 Alberta Canada

**Keywords:** breed composition, breed diversity, *Capra hircus*, heterozygosity, indigenous goats, population genetics

## Abstract

Uganda has a large population of goats, predominantly from indigenous breeds reared in diverse production systems, whose existence is threatened by crossbreeding with exotic Boer goats. Knowledge about the genetic characteristics and relationships among these Ugandan goat breeds and the potential admixture with Boer goats is still limited. Using a medium‐density single nucleotide polymorphism (SNP) panel, we assessed the genetic diversity, population structure and admixture in six goat breeds in Uganda: Boer, Karamojong, Kigezi, Mubende, Small East African and Sebei. All the animals had genotypes for about 46 105 SNPs after quality control. We found high proportions of polymorphic SNPs ranging from 0.885 (Kigezi) to 0.928 (Sebei). The overall mean observed (H_*O*_) and expected (H_*E*_) heterozygosity across breeds was 0.355 ± 0.147 and 0.384 ± 0.143 respectively. Principal components, genetic distances and admixture analyses revealed weak population sub‐structuring among the breeds. Principal components separated Kigezi and weakly Small East African from other indigenous goats. Sebei and Karamojong were tightly entangled together, whereas Mubende occupied a more central position with high admixture from all other local breeds. The Boer breed showed a unique cluster from the Ugandan indigenous goat breeds. The results reflect common ancestry but also some level of geographical differentiation. admixture and f_4_ statistics revealed gene flow from Boer and varying levels of genetic admixture among the breeds. Generally, moderate to high levels of genetic variability were observed. Our findings provide useful insights into maintaining genetic diversity and designing appropriate breeding programs to exploit within‐breed diversity and heterozygote advantage in crossbreeding schemes.

## Introduction

According to archaeo‐zoological evidence, goats were among the first ungulates to be domesticated, about 10 000 years ago near the fertile crescent that spans from Eastern Anatolia to the Zagros Mountains in Northern Iran (Zeder & Hesse 2000; Naderi *et al*. 2008). Archaeological evidence suggests the rapid spread of goats from the centre of domestication to Eurasia and Africa following human migrations and trade routes. Migration of goats into Africa occurred through three main entry routes: one along the Mediterranean coast, a second via the Red Sea hills region and a third through the Nile Valley via the Sinai peninsula and the Nile delta (Taberlet *et al*. 2008; Pereira *et al*. 2009; Gifford‐Gonzalez & Hanotte 2011). Other movements have also been reported from the Near East into the Ethiopian highlands and central Sahara (Clutton‐Brock 2000).

Today, goats are among the most important livestock species in developing countries. They are of significant socio‐economic, nutritional and cultural importance in smallholder farming systems. Uganda has three major indigenous goat breeds (Mubende, Kigezi and Small East African goats) that are geographically isolated and are raised in diverse production systems (Mason & Maule 1960; Nsubuga 1996; MAAIF & UBOS 2009). Besides these three indigenous breeds, several indistinct ecotypes of Ugandan indigenous goat breeds exist, including Karamojong and Sebei. In the early 1990s, crossbreeding with Boer goats introduced from South Africa was initiated to improve the production characteristics of the Ugandan indigenous goats (Nsubuga 1996). Boer goats are widely used as a source of breeding stock to cross with the indigenous goats (Onzima *et al*. 2014). The choice of Boer goats was premised on the fact that they have a fast growth rate and exhibit better disease resistance than do other exotic goat breeds (Casey & Van Niekerk 1988). However, with uncoordinated breeding management, indiscriminate crossing may occur, increasing the risk of the disappearance of resilient and well‐adapted indigenous breeds. The existence of the various breeds presents an enormous source of diversity in the current goat populations that needs to be characterized, conserved and utilized in a sustainable manner under the existing production systems. Genetic diversity in populations is important, as it provides the basis for natural as well as artificial selection (Qanbari & Simianer 2014).

In order to study diversity, molecular tools are essential as a valuable complement to the evaluation of phenotypes and production systems and, sometimes, as a proxy for phenotypic diversity of local breeds (Ajmone‐Marsan *et al*. 2014). However, compared to other livestock species, African goats remain poorly studied, especially at the molecular level. Earlier studies in Africa, using mitochondrial and microsatellite DNA markers, indicate a lack of phylogeographic structure among the goat breeds (Alemu 2004; Chenyambuga *et al*. 2004; Okpeku *et al*. 2011; Hassen *et al*. 2012; Benjelloun *et al*. 2015). These studies were geared mainly towards assessing genetic diversity in an attempt to monitor genetic erosion and to identify conservation priorities. In Uganda, earlier genetic characterization of indigenous goats was carried out using a limited number of microsatellites (Chenyambuga *et al*. 2004; Muema *et al*. 2009). A drawback of microsatellite analysis is that it is difficult to integrate data across laboratories, due mainly to the inherent poor reproducibility of allele calling (FAO 2011). Therefore, a comparison of results from different studies that used microsatellites is complicated.

However, the advent of the GoatSNP50 BeadChip in 2014 (Tosser‐Klopp *et al*. 2014) has changed the landscape and depth of genomic research in goats (Tosser‐Klopp 2015) due to its robustness, low genotyping costs, automatic allele calling and ability to interrogate the goat genome at high resolution (Ajmone‐Marsan *et al*. 2014). The Illumina GoatSNP50 BeadChip, which features 53 347 single nucleotide polymorphisms (SNPs), was developed from SNP loci detected by whole genome sequencing of six goat breeds (Tosser‐Klopp *et al*. 2014; Tosser‐Klopp 2015). The SNP chip has been used to study genetic diversity and population structure of goats in various countries with indigenous goat breeds in locales such as Italy (Nicoloso *et al*. 2015), Spain (Manunza *et al*. 2016), South Africa (Lashmar *et al*. 2016; Mdladla *et al*. 2016; Visser *et al*. 2016), Ethiopia (Abegaz 2014; Mekuriaw 2016) and Australia (Kijas *et al*. 2013). Therefore, the objective of the current study was to: (i) assess the degree of genetic diversity in Ugandan goat breeds using SNPs; (ii) infer population structure and breed relationships; and (iii) investigate admixture among breeds, namely the influence of the commercial Boer breed in Ugandan goats. The information generated from this study can be used in management and conservation of Ugandan goat genetic resources and makes it possible to design effective strategies for breed improvement.

## Materials and methods

### Animal resources and sampling

A total of 144 animals from six goat breeds were included in this study. Five indigenous goat breeds (Mubende, Kigezi, Small East African, Karamojong and Sebei) were sampled from 79 smallholder farms/herds, and the exotic Boer goats were sampled from a commercial multiplication centre (Ssembeguya Estates) and a government breeding centre (Rubona Stock farm), which are sources of breeding stock for goat improvement in Uganda.

**Figure 1:**
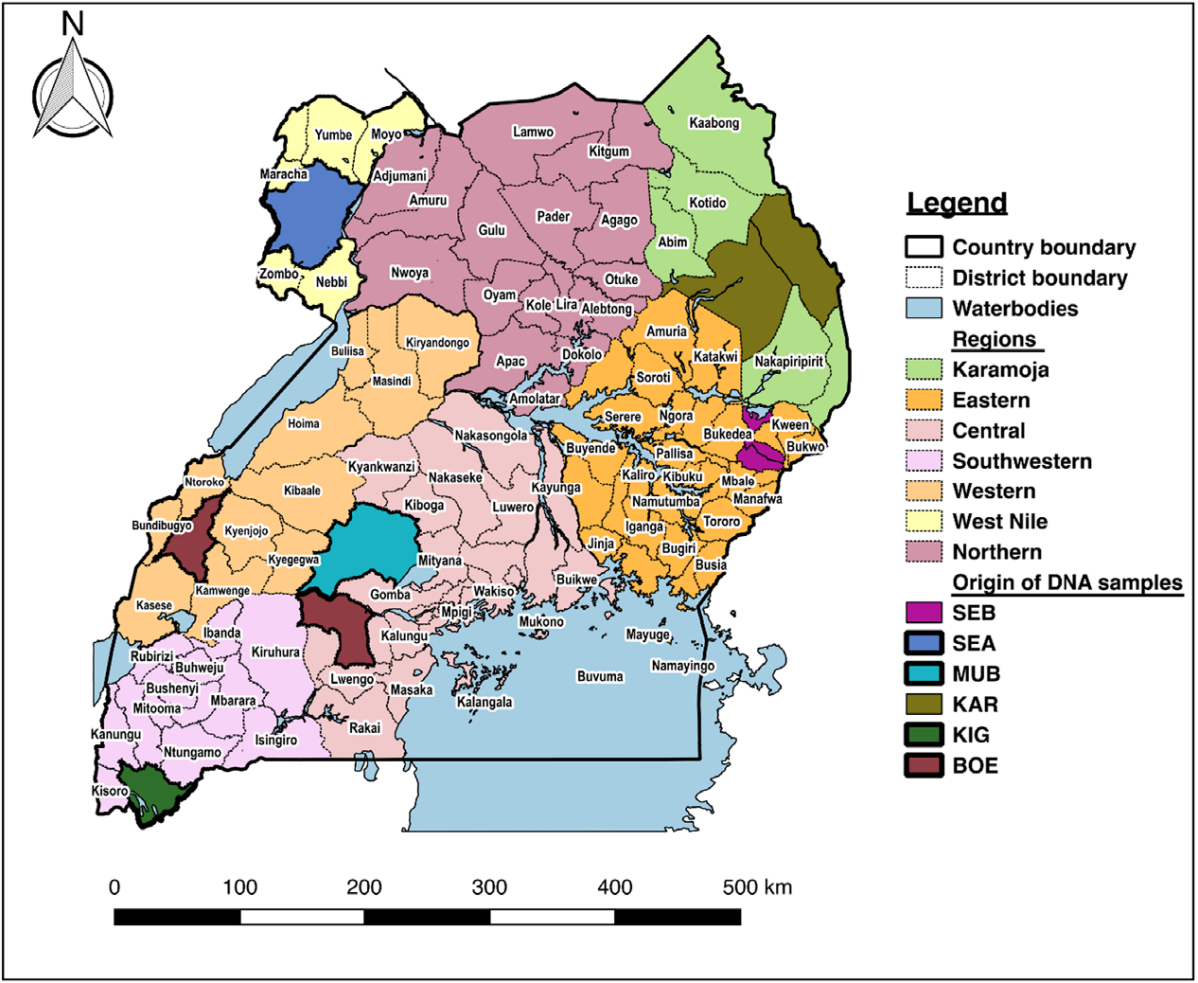
Map of Uganda showing geographical origin of the goat DNA samples analysed. Breed acronyms are defined as follows: BOE, Boer; KAR, Karamojong; KIG, Kigezi; MUB, Mubende; SEA, Small East African; SEB, Sebei.

Sampling was carried out at selected geographical locations (Fig. 1) according to livestock statistics from the Livestock Census Report 2008 (MAAIF & UBOS 2009). The goat populations sampled originated from the following five agro‐ecological zones of Uganda: Mubende goats from Mubende district in the mid‐altitude farmlands and central wooded savanna, Kigezi from Kabale and Kisoro districts in the southwestern highlands, Small East African from Arua district in the short savanna grasslands, Karamojong from Moroto district in the northeastern semi‐arid region, Sebei from Sironko in the eastern highlands and Boer goats from Ssembabule and Kabarole districts in the mid‐altitude zone. Sampling was conducted to cover a wide distribution of individual animals across the selected production locations. Within a herd, we relied on the farmers’ pedigree knowledge to select, as much as possible, unrelated individuals.

Ear punch tissue was collected from the 144 goats at smallholder farms for the indigenous breeds Mubende (*n *=* *29), Kigezi (*n *=* *29), Small East African (*n *=* *29), Karamojong (*n *=* *15) and Sebei (*n *=* *29) and at a commercial and a government breeding centre for Boer (*n *=* *13). The ear tissue samples were collected into vials containing a desiccant and stored within 12 h in a freezer at −4 °C.

The study was approved by the Ethics Committee of Uganda National Council of Science and Technology (UNCST; SBLS/REC/15/131).

### DNA extraction

Genomic DNA was extracted using the DNeasy blood and tissue kit (Qiagen^®^). Twenty DNA samples were randomly selected and analyzed on a 1% agarose gel for a preliminary estimate of the DNA quality and quantity. The final DNA quality and quantity were validated using the Qubit® dsDNA BR (Broad‐Range) Assay Kit on the Qubit 2.0 fluorimeter (Invitrogen) prior to genotyping.

### Genotyping and quality control

DNA samples were genotyped with the Illumina GoatSNP50 BeadChip. The BeadChip, developed by the International Goat Genome Consortium (IGGC), features 53 347 SNPs across the whole goat genome with inter‐SNP spacing of approximately 40 kb (Tosser‐Klopp *et al*. 2014). Data were analyzed using genome studio ^™^ software v1.1 (Illumina, Inc.). Genomic locations of the SNPs and cluster files were provided by IGGC. Standard SNP genotype quality control procedures were performed using plink v1.07 (Purcell *et al*. 2007). Individuals with a missing genotype call rate of greater than or equal to 10% were excluded from further analysis using the –*mind* function in plink. The remaining individuals were then subjected to SNP quality control. SNPs with a call rate of less than 0.95, minor allele frequency (MAF) of 0.05 or less and SNPs whose genotypes were not in Hardy Weinberg equilibrium (*P *<* *0.001) were excluded from downstream analysis. The dataset of SNPs used in the analysis is available from https://www.animalgenome.org/repository/pub/WAGNL2017.1002/.

### Data analysis

The observed (H_*O*_) and expected (H_*E*_) heterozygosities for the respective populations were calculated using plink (Purcell *et al*. 2007). The population structuring and relatedness were estimated from the SNP genotypes using principal components analysis (PCA), available from the r package snprelate (Zheng *et al*. 2012).

Additionally, population structure analyses were performed to infer the most likely number of ancestral populations using admixture software version 1.23 (Alexander *et al*. 2009; Alexander & Lange 2011). To estimate the individual ancestry within the population, admixture employs prior defined *K* values corresponding to the assumed number of ancestral populations. The procedure involves the use of maximum likelihood estimates on data from multiple loci to estimate individual ancestry within the population being considered. To determine the most optimal population structure, a cross‐validation procedure was undertaken with hypothetical admixture runs from *K *=* *2 to 7. Optimal partitioning of the population was achieved at the lowest cross‐validation error.

Phylogenetic relationships between the goat breeds were inferred using the Neighbour‐Net procedure in splitstree 4 software (Huson & Bryant 2006) based on Reynold's genetic distances, whereas individual relationships across all breeds were calculated using identity‐by‐state distances.

To further investigate admixture in Ugandan goat breeds, we performed a three‐population (f_3_) test (Reich *et al*. 2009; Patterson *et al*. 2012) and a four‐population (f_4_) test (Keinan *et al*. 2007; Patterson *et al*. 2012) implemented in treemix (Pickrell & Pritchard 2012). These statistics are used to explain admixture history of the populations being investigated, particularly when correlations in allele frequencies do not conform to population evolution with a split tree (Reich *et al*. 2009; Patterson *et al*. 2012). To provide support for past admixture events between the populations, the threepop program from TreeMix was used to calculate f_3_ (A;B,C) statistics for all possible combinations of three populations. Generally, if population A is a result of an admixture between two other populations B and C, the calculated *z*‐score for each tested combination of three populations would have a significant negative value. A positive *z*‐score may indicate either absence of admixture or substantial post‐admixture drift resulting from the alleles in the population. Meanwhile, the fourpop program from TreeMix was used to calculate f_4_ (A,B; C,D) statistics for subsets of the population. The four‐population test f_4_ (A,B; C,D) (Keinan *et al*. 2007; Patterson *et al*. 2012) was used to test if A,B and C,D were genetically distinct groups (clades) in the population tree. A significant non‐zero *z*‐score indicates gene flow between A,B and C,D in the population tree (Keinan *et al*. 2007; Patterson *et al*. 2012; Makina *et al*. 2016). Larger values indicate strong evidence of gene flow in the tree.

## Results

### Level of SNP polymorphism within breeds

After quality control procedures on the 53 347 SNPs included in the SNP chip, 7242 SNPs were excluded (Table 1), which resulted in 46 105 loci available for downstream analysis. Of the SNPs excluded, 2093 showed a SNP call rate of less than 0.95, 3500 had MAFs less than 0.05 and 2817 significantly deviated from Hardy–Weinberg equilibrium (*P *<* *0.001). The highest number of SNPs that showed a MAF of less than 0.05 was found in Small East African (*n *=* *7818), whereas Sebei showed the lowest number of SNPs excluded (*n *=* *6826). All animals passed the quality criteria and were used in the analysis.

**Table 1:** Number of animals and SNPs excluded and remaining after quality control procedures on genotype data

Breed	*n*	Excluded SNPs^a^ SNP CR < 0.95	MAF < 0.05	HWE	Total	Remaining SNPs
Boer	13	2577	5280	208	7323	46 024
Karamojong	15	2640	5687	358	7803	45 544
Kigezi	29	1977	6139	543	7793	45 554
Mubende	29	2260	4922	767	7034	46 313
Small East African	29	2523	5669	589	7818	45 529
Sebei	29	2429	4824	469	6826	46 521
Merged	144	2093	3500	2817	7242	46 105

*N,* number of animals; CR, call rate; MAF, minor allele frequency; HWE, chi square test for Hardy‐Weinberg equilibrium (*P*‐value < 0.001).

a Some SNPs were excluded due to more than a single criterion.

**Figure 2:**
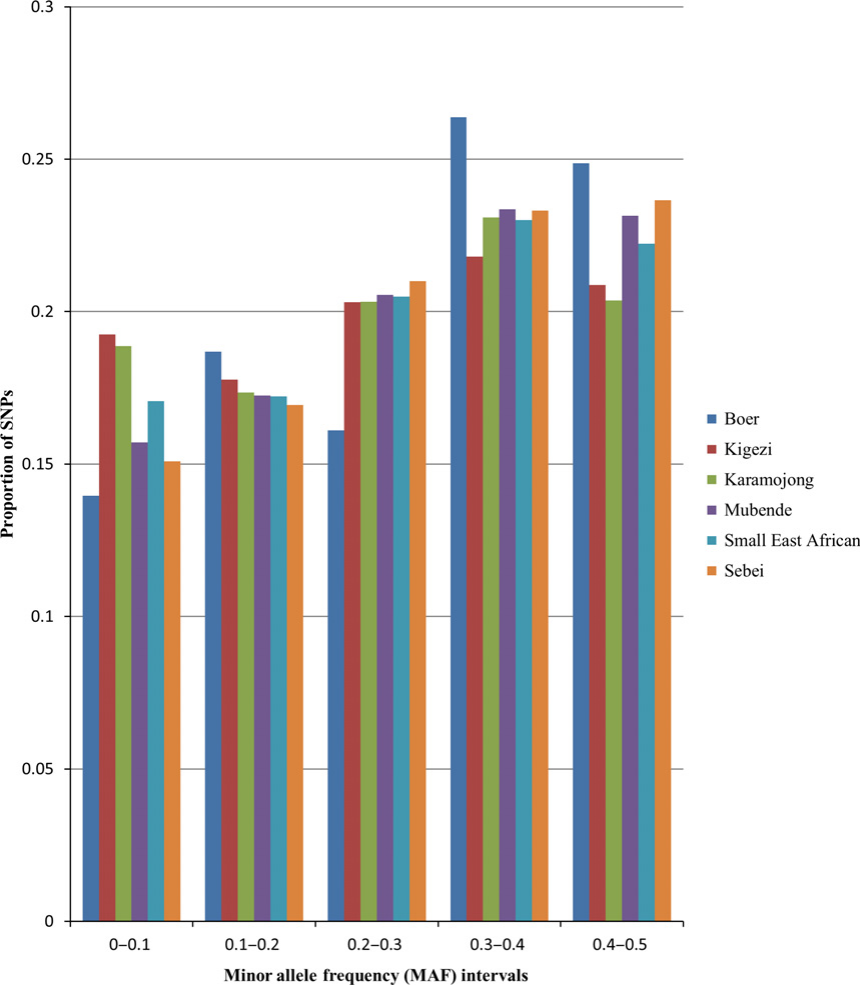
Distribution of SNPs by MAF intervals in each breed.

Boer (0.51) and Kigezi (0.43) showed the highest and lowest proportion of SNPs with MAF greater than or equal to 0.3 respectively (Fig. 2). The proportion of fixed loci (MAF = 0) was similar across the breeds, ranging from Karamojong (0.06) to Mubende (0.04). Despite only Boer being represented in the group of goat breeds used to develop this SNP array, 93.4% of the SNP markers across the six goat breeds was polymorphic (MAF ≥ 0.05) (Table 2). The highest proportion of polymorphic loci (P_*N*_) was found in Sebei (0.928) and the lowest in Kigezi (0.885); however, the differences in P_*N*_ were negligible across all the breeds.

**Table 2:** Population characteristics showing proportion of polymorphic SNPs (P_*N*_)*,* mean minor allele frequency (MAF), expected (H_*E*_) and observed (H_*O*_) heterozygosity for the six goat populations

Breed	*n*	P_*N*_	MAF	H_*E*_ * *±* *SD	H_*O*_ * *± SD
Boer	13	0.901	0.280	0.408 ± 0.178	0.377 ± 0.193
Karamojong	15	0.893	0.271	0.410 ± 0.192	0.357 ± 0.192
Kigezi	29	0.885	0.257	0.377 ± 0.189	0.340 ± 0.181
Mubende	29	0.908	0.272	0.391 ± 0.179	0.355 ± 0.178
Small East African	29	0.894	0.266	0.393 ± 0.189	0.349 ± 0.180
Sebei	29	0.928	0.274	0.395 ± 0.178	0.365 ± 0.176
Merged	144	0.934	0.289	0.384 ± 0.143	0.355 ± 0.147

### Breed genetic diversity

Genetic diversity was assessed within each breed (Table 2). The results indicate small differences in genetic diversity between the breeds. The lowest observed heterozygosity was found in Kigezi (H_*O*_ = 0.340 ± 0.181) and the highest in Boer (H_*O*_ = 0.377 ± 0.193), indicating higher diversity in Boer compared to Kigezi. Also, the MAFs across all loci were lowest in Kigezi (0.257) and highest in Boer (0.280). In general, the observed heterozygosity was slightly lower than the expected heterozygosity (H_*O*_ *< *H_*E*_), indicating a deficiency in heterozygosity across all the breeds.

### Population structure analysis

**Figure 3:**
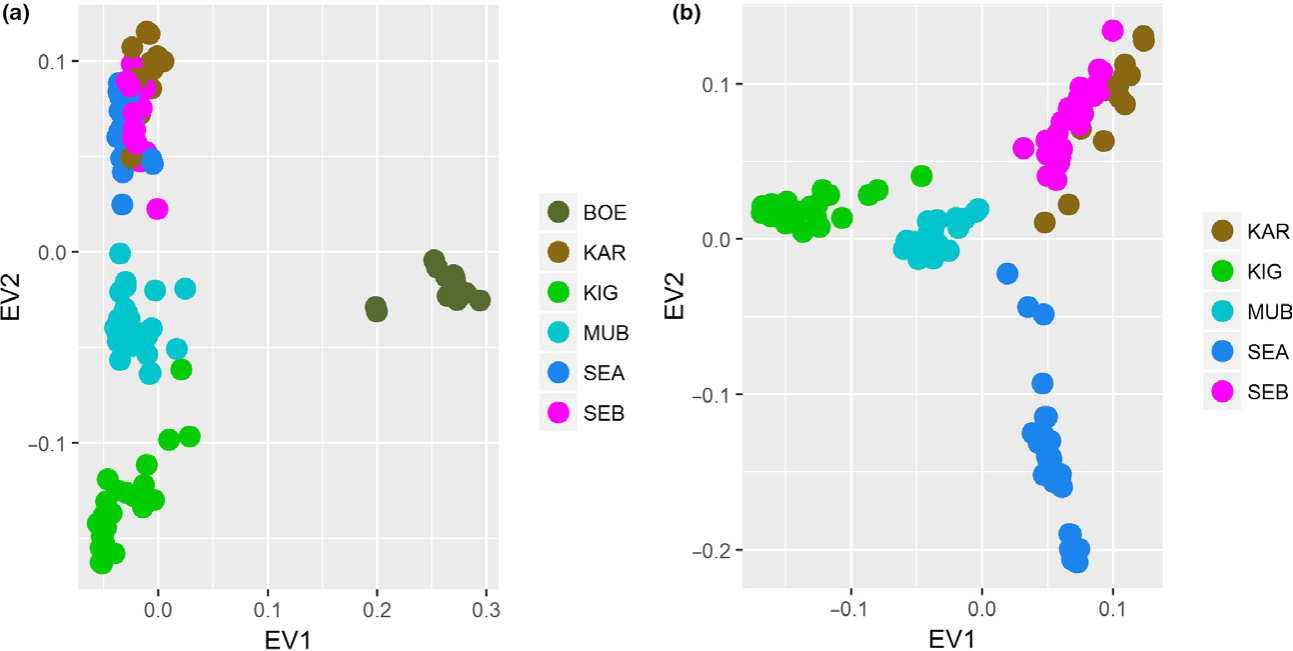
Plot of two principal components showing genetic relationships among: (a) five Ugandan indigenous goats and Boer breeds and (b) five Ugandan indigenous goat breeds only. Goat populations analysed: BOE, Boer; KAR, Karamojong; KIG, Kigezi; MUB, Mubende; SEA, Small East African; SEB, Sebei.

The first principal component, eigen vector 1 (EV1) shown in Fig. 3a, separated Boer from the Ugandan indigenous goat breeds and accounted for 10.7% of the total variance. The second principal component (EV2) accounted for 3.2% of the total variance and divided the Ugandan indigenous goat breeds into two clusters: a distinct cluster comprising Kigezi and Mubende breeds and a combined breed cluster consisting of Sebei, Karamojong and Small East African breeds (Fig. 3). A more detailed analysis, in which the first principal component (EV1) explained 3.6% and the second principal component (EV2) accounted for 2.9% of total variance in the Ugandan indigenous goat breeds, showed a similar clustering pattern for all breeds except the Small East African goats, which formed a separate cluster (Fig. 3b). Mubende clustered among all other breeds, indicating possible admixture with the other goats.

**Figure 4:**
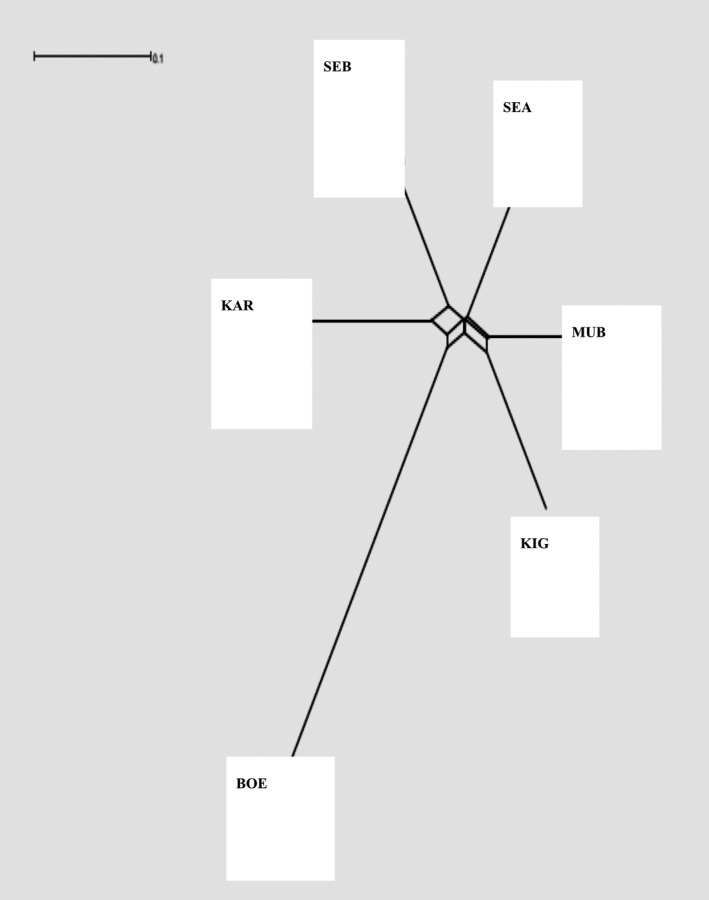
Neighbour‐Net graph based on Reynolds genetic distances depicting breed relationships among five Ugandan indigenous goat populations and one commercial goat breed. BO, Boer; KAR, Karamojong; KIG, Kigezi; MUB, Mubende; SEA, Small East African; SEB, Sebei.

Breed relationships were also assessed by computing genetic distances between each pair of individuals from the number of loci for which they differ. Based on the estimated genetic distances, a Neighbour‐Net graph was computed to depict breed clustering (Fig. 4). The Ugandan indigenous breeds showed short branching, suggesting low differentiation between the breeds, whereas the exotic Boer goat breed showed a long branch, suggesting a well‐differentiated and distinctive clade. Individuals belonging to the same breed mostly clustered together, as inferred by the identity‐by‐state distance‐based neighbour‐joining (NJ) tree (Fig. S1). Some Sebei and Karamojong individuals appeared to be entangled (or admixed), whereas Mubende was sub‐divided into two groups. The remaining breeds (i.e. Small East African, Kigezi and Boer) all formed tight groups.

### Genetic admixture

**Figure 5:**
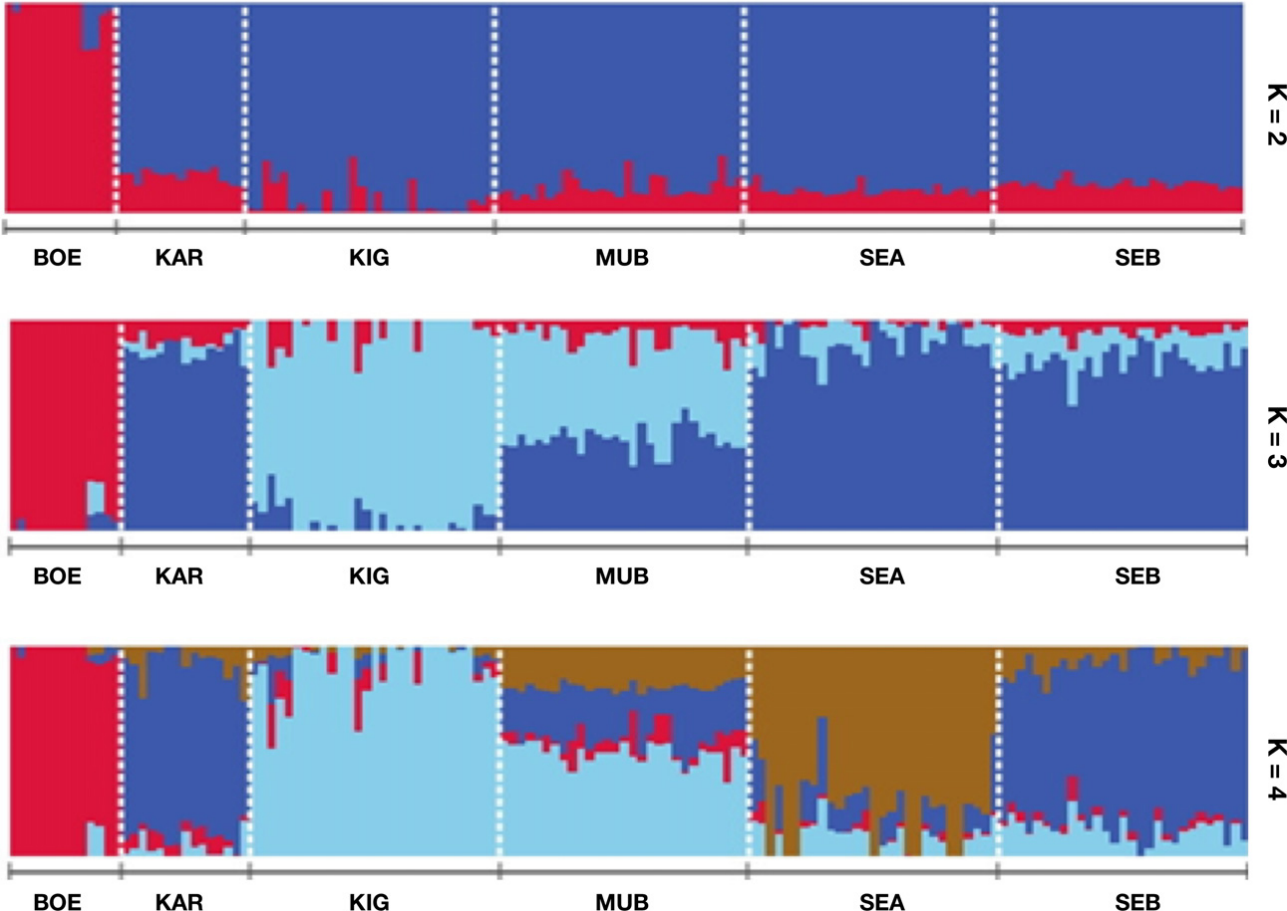
Population structure plots showing proportions of ancestral populations for each individual for *K *=* *2 to 4. BOE, Boer; KAR, Karamojong; KIG, Kigezi; MUB, Mubende; SEA, Small East African; SEB, Sebei.

Similar to the results from PCA, admixture analysis at *K *=* *2 separated the Ugandan indigenous goats from the commercial Boer goats (Fig. 5). Additionally, at *K *=* *3 the analysis separated the populations into three subpopulations: Boer; Kigezi and Mubende; and Small East African, Karamojong and Sebei; it also indicated a considerable component of Boer and Kigezi in the subpopulations. Based on the least cross‐validation error (Fig. S2), *K *=* *4 was identified as the optimal number of ancestral populations and indicated a Boer component in all five Ugandan indigenous goat breeds. On average, around 3, 5, 5, 1 and 1% of the Boer goat genome was shared with Karamojong, Kigezi, Mubende, Small East African and Sebei goats respectively (Table 3). The analysis also revealed a finer resolution of the Ugandan indigenous breeds, by which Kigezi and Small East African goats emerged as a distinct groups, Karamojong and Sebei remained tightly clustered together and the Mubende breed appeared to be the more admixed population, comprising Kigezi, Small East African and Karamojong/Sebei breeds (50%, 20% and 25% respectively). admixture runs from *K *=* *2 to 4 revealed considerable admixture among the breeds, and gene flow from Boer was observed in all the indigenous Ugandan goat breeds.

**Table 3:** Average breed composition of six goat populations given four clusters estimated by admixture software

Breed	*n*	Cluster 1 (SEA)	Cluster 2 (KAR/SEB)	Cluster 3 (BOE)	Cluster 4 (KIG)
Boer	13	0.008 ± 0.017	0.018 ± 0.027	0.950 ± 0.084	0.024 ± 0.055
Karamojong	15	0.091 ± 0.079	0.840 ± 0.120	0.031 ± 0.025	0.038 ± 0.056
Kigezi	29	0.022 ± 0.027	0.027 ± 0.046	0.045 ± 0.070	0.905 ± 0.129
Mubende	29	0.202 ± 0.022	0.245 ± 0.066	0.049 ± 0.053	0.503 ± 0.055
Small East African	29	0.776 ± 0.178	0.123 ± 0.107	0.009 ± 0.023	0.091 ± 0.070
Sebei	29	0.077 ± 0.059	0.784 ± 0.121	0.012 ± 0.021	0.127 ± 0.058

BOE, Boer; KAR, Karamojong; KIG, Kigezi; MUB, Mubende; SEA, Small East African; SEB, Sebei goats.

To further confirm admixture among the goat breeds, we calculated f_3_ statistics for all possible three‐population groups for all six breeds and f_4_ statistics for all possible sub‐populations for sister and opposing sister groups. With the Ugandan goats in this study, we found only four significant f_3_ tests, all of which involved the Mubende breed (Table 4). This suggests admixture between Mubende and the other goat breeds.

**Table 4:** Summary of three‐population tests with significant f_3_ statistics showing admixture in Ugandan goats with Kigezi as one of the source populations

Population A (admixed)	Population B (source)	Population C (source)	f_3_ statistic	SE	*z‐*score
MUB	BOE	KIG	−0.0017	0.0002	−7.1891^a^
MUB	KIG	SEB	−0.0012	0.0001	−13.7787^a^
MUB	KAR	KIG	−0.0016	0.0001	−16.0069^a^
MUB	KIG	SEA	−0.0016	0.0001	−19.4077^a^

BOE, Boer; KAR, Karamojong; KIG, Kigezi; MUB, Mubende; SEA, Small East African; SEB, Sebei goats.

a Significant f_3_ statistics (*P *<* *0.05).

Based on the f_4_ test statistic, combining Boer with any of the Ugandan indigenous goats resulted in the most significant values (Table 5). This suggests gene flow from Boer into Ugandan indigenous breeds. Similarly, the significant f_4_ statistics for subpopulations involving Karamojong and Kigezi goats with the other Ugandan indigenous goats suggest gene flow from these breeds (Table S1).

**Table 5:** Summary of four‐population tests showing some significant f_4_ statistics to detect admixture and gene flow within Ugandan indigenous and Boer goat breeds

Population 1	Population 2	Population 3	Population 4	f_4_ statistic	SE	*z*‐score
BOE	KIG	KAR	MUB	0.0050	0.0002	20.5959^a^
BOE	MUB	KAR	KIG	0.0051	0.0003	20.4125^a^
BOE	KAR	MUB	SEB	0.0034	0.0002	16.4090^a^
BOE	KIG	KAR	SEB	0.0022	0.0002	14.3987^a^
BOE	KAR	KIG	SEB	0.0033	0.0003	12.5043^a^
BOE	MUB	KAR	SEB	0.0018	0.0002	12.1647^a^
BOE	MUB	KAR	SEA	0.0027	0.0003	10.8053^a^
BOE	KIG	KAR	SEA	0.0027	0.0003	10.2101^a^
BOE	KAR	SEA	SEB	0.0020	0.0002	9.2824^a^
BOE	SEA	KAR	SEB	0.0014	0.0002	8.2888^a^
BOE	KAR	MUB	SEA	0.0014	0.0002	6.0396^a^
BOE	SEA	KAR	MUB	0.0013	0.0002	5.6349^a^
BOE	SEA	KAR	KIG	0.0014	0.0003	5.0805^a^
BOE	SEB	MUB	SEA	0.0010	0.0002	4.4690^a^
BOE	KAR	KIG	SEA	0.0013	0.0003	4.4078^a^
BOE	SEB	KAR	KIG	−0.0011	0.0003	−4.0038^a^
BOE	MUB	SEA	SEB	−0.0009	0.0002	−4.1880^a^
BOE	SEB	KAR	MUB	−0.0016	0.0002	−6.4749^a^
BOE	KIG	MUB	SEA	−0.0023	0.0002	−9.8169^a^
BOE	MUB	KIG	SEA	−0.0024	0.0003	−9.4555^a^
BOE	KIG	MUB	SEB	−0.0028	0.0002	−12.9529^a^
BOE	MUB	KIG	SEB	−0.0033	0.0002	−14.3171^a^

BOE, Boer; KAR, Karamojong; KIG, Kigezi; MUB, Mubende; SEA, Small East African; SEB, Sebei goats.

a Significant f_4_ statistics indicating presence of gene flow.

## Discussion

In this study, we assessed genetic diversity, population structure and admixture in Ugandan indigenous goat breeds at a genome‐wide scale using a moderately dense SNP panel. We further assessed the presence of admixture of an exotic goat breed (Boer) into the Ugandan breeds.

### Genotypic data and level of polymorphism

The first objective of the study was to assess the level of polymorphism in Ugandan goat breeds using the GoatSNP50 BeadChip. Our results show that the proportion of polymorphic loci within Ugandan goat breeds ranges from 0.885 in Kigezi goats to 0.928 in Sebei (Table 2). This high level of genetic polymorphism indicates that most of the SNPs are segregating in the breeds under investigation. The level of polymorphism observed in this study is similar to those observed during SNP discovery and validation within breeds with similar numbers of animals genotyped (Tosser‐Klopp *et al*. 2014). The GoatSNP50 BeadChip was developed using dairy and mixed breeds (Alpine, Saanen and Creole) and meat‐type breeds (Boer, Katjang and Savanna). The chip was validated with 10 breeds from different backgrounds. The SNPs were segregating at greater than 78% in seven of the breeds, including Angora and Skopelos, which were not used during SNP discovery (Tosser‐Klopp *et al*. 2014). Similar levels of polymorphism in goat breeds have been reported elsewhere (Kijas *et al*. 2013; Nicoloso *et al*. 2015; Lashmar *et al*. 2016; Mdladla *et al*. 2016; Mekuriaw 2016). For example, Mdladla *et al*. (2016) reported levels of polymorphism ranging from 84.2 to 97.6% in nine South African indigenous goats, 96.8 to 99.7% in Italian goats (Nicoloso *et al*. 2015) and greater than 97% in Australian goat breeds (Kijas *et al*. 2013). The success of the chip can be attributed to the use of six goat breeds from different types, origins and production environments for SNP discovery. Therefore, similar high levels of polymorphism were envisaged across other breeds that were not used during the design of the SNP chip.

### Breed genetic diversity

The Ugandan indigenous goat and Boer breeds show a high degree of genetic diversity, as determined by the high heterozygosity values detected in this study. Our results revealed that the expected and observed heterozygosities ranged from H_*E*_ = 0.377 ± 0.189 (Kigezi) to H_*E*_ = 0.410 ±0.192 (Karamojong) and H_*O*_ = 0.340 ± 0.181 (Kigezi) to H_*O*_ = 0.377 ± 0.193 (Boer) respectively (Table 2). The heterozygosity values obtained in this study are comparable with those reported for indigenous goats in Ethiopia (Mekuriaw 2016), South Africa (Mdladla *et al*. 2016), Egypt (Kim *et al*. 2016), Spain (Manunza *et al*. 2016) and Italy (Nicoloso *et al*. 2015) as well as for commercial goats from Canada and Australia (Brito *et al*. 2017); South Africa (Lashmar *et al*. 2016); and a variety of Angora goat populations in Argentina, France and South Africa (Visser *et al*. 2016).

The expected and observed heterozygosity for Boer (H_*E*_ = 0.408 ± 0.178; H_*O*_ = 0.377 ± 0.193 respectively) were slightly higher than those reported for Boer populations in Canada (H_*E*_ = 0.357; H_*O*_ = 0.363 respectively) (Brito *et al*. 2017) and Australia (H_*E*_ = 0.355; H_*O*_ = 0.363 respectively) (Kijas *et al*. 2013). These differences may be attributed to differences in effective population sizes, duration of isolation and selection practices in the different production systems.

We obtained the highest expected heterozygosity in Karamojong goats, which could be due to the pastoral production system used. Under communal production systems practiced by pastoral and smallholder farmers, there is an absence of structured artificial selection programs, with random mating and high admixing between populations and herds probably occurring. This favours an increase in genetic variability and reduction in inbreeding, which is a decisive factor in the success for conservation programs. A similar trend of genetic diversity was also reported for indigenous goat breeds in South Africa (Mdladla *et al*. 2016) and local goat breeds in Brazil (da Rocha *et al*. 2016). Similarly, a study investigating breed composition of Creole goats from 10 American countries found moderate to high heterozygosity values (Ginja *et al*. 2017).

The difference between the observed and expected heterozygosity was small and within a fraction of one standard error. As a general trend, the observed heterozygosity was lower than the expected heterozygosity (H_*O*_ *< *H_*E*_) within all breeds. Thus, these differences may be due to a Wahlund effect rather than inbreeding.

### Population structure and admixture

The results of the population structure and admixture analyses indicate that the five Ugandan goat breeds are weakly differentiated. This may be due to the recent establishment of these breeds from probably the same founder population or related populations, but to confirm this, an in‐depth analysis of the breed history will be required. Another possible explanation for the low degree of differentiation could be continuous gene flow between the indigenous breeds.

The results of our population structure and admixture analyses are in agreement. The three methods were used separate the breeds into clusters. The first principal component separates the Boer from the Ugandan indigenous goat breeds (Fig. 3). Furthermore, the presence of a distinct branching of Boer in the NJ tree suggests a differentiated gene pool (Fig. 4).

At *K *=* *2, the admixture analysis separates the breeds into two distinct clusters: the Boer and the Ugandan indigenous goats (Fig. 5). This observation is in agreement with the PCA results, which showed the same two major clusters. Optimal clustering is observed at *K *=* *4, a value at which the cross‐validation error is lowest (Fig. S2). At this optimal *K* value, admixture analysis differentiates Ugandan indigenous goat breeds into distinct clusters of Small East African/Kigezi and Karamojong/Sebei tightly grouped together, whereas Mubende is admixed showing influences from the three groups. The differentiation of Kigezi and Small East African goats could be attributed to genetic drift but could also have resulted from selection and/or adaptation pressures.

The three‐ and four‐population tests (Keinan *et al*. 2007; Reich *et al*. 2009; Patterson *et al*. 2012) were used to further qualify admixture and gene flow. Using the three‐population (f_3_) test statistic, we found strong evidence of admixture in only four comparisons, all involving the Mubende goats as the admixed breed. The most significant *z*‐score (−19.408) was found with the Mubende f_3_ (Mubende/Kigezi/Small East African). All the significant f_3_ statistics were observed when Kigezi was one of the source populations, indicating that Kigezi might be contributing to the gene pool either through ancestral generations or crossbreeding with Mubende. The f_4_ statistics showed the most significant scores for the Boer and the Ugandan indigenous goats (Table S1). This is supported by the results of the admixture analysis, as shown by the breed composition of the individual goats studied (Table S2). However, determining the extent of the admixture in the Ugandan goat populations requires further studies involving larger sample sizes and more ecotypes and breeds.

The indigenous Ugandan goat breeds showed clear differentiation according to their geographical regions. The results show a clear differentiation of Kigezi and Small East Africa, whereas Karamojong and Sebei remained tightly clustered together, which may be attributed to the contiguous territory of the breeds (Fig 1). Mubende is centrally located and prone to admixture with Kigezi, Karamojong/Sebei and Small East African. The lack of differentiation in some of the indigenous breeds is also confirmed by the presence of short branches in the NJ tree (Fig. 4), suggesting a high level of genetic similarity and low divergence, which may be attributed to local admixture between the breeds. The lack of differentiation in geographically diverse populations may also indicate common ancestry, short domestication history and lack of selection pressure, and the mobility of the goats may also play a role. The Boer goat breed was introduced into Uganda for improving growth characteristics of the indigenous goats for meat production. The breed originates from South Africa, where it has been extensively selected for faster growth (Casey & Van Niekerk 1988).

Our results support admixture among all the breeds, although the highest admixture was observed in Mubende goats. This may be due to the fact that the breed is widely used across production systems as a preferred breed by the farmers due to its large live body weight (Onzima *et al*. 2016, 2017). admixture analysis further revealed admixing between the Boer and the indigenous goats; however, the results suggest there is limited gene flow from Boer to the Ugandan indigenous goat populations due to crossbreeding.

## Conclusion

Overall, the results described in this study indicate high genetic variability of the Ugandan goat populations and sufficient genetic potential for further improvement of the breeds for heritable economic traits. The Ugandan indigenous goats are weakly differentiated, consisting of two breeds forming more uniform clusters (Kigezi and Small East African), two breeds clearly crossbred (Karamojong and Sebei) and Mubende showing signs of gene flow from all these goat populations. Nonetheless, there is rather limited Boer admixture in the Ugandan goat population. This knowledge can be exploited to devise strategies for sustainable utilization and maintenance of genetic diversity.

## Conflict of interest

The authors declare that they have no conflict of interest whatsoever to influence the outcome of the manuscript.

## Authors’ contributions

RBO conceived and designed the study, collected the field samples and wrote the manuscript. MAMG, EK and RPMAC supervised the study. MRU, RBO and RM participated in data analysis. All authors have read and edited the manuscript.

## Supporting information


**Figure S1** Genetic relationships among five Ugandan indigenous goat breeds and one commercial goat breed; constructed using a neighbour‐ joining tree from identity‐by‐state (IBS) distances derived from 46 105 SNPs.

**Figure S2** Cross‐validation error plot indicating the choice of the appropriate *K* value**.**

**Table S1** Significant f_4_ statistics for Ugandan goat breeds indicating gene flow in the breeds.

**Table S2** Breed composition of cluster 1, 2, 3 and 4, interpreted as representative of Small East African, Karamojong/Sebei, Boer and Kigezi ancestries respectively, estimated for each individual belonging to goat populations from Uganda.
