# Supplementary material for: Genome‐wide population structure and admixture analysis reveals weak differentiation among Ugandan goat breeds
Source: Anim Genet. 2018 Jan 17;49(1):59–70. doi: 10.1111/age.12631 (PMC5838551; doi:10.1111/age.12631)

Figure S1 Genetic relationships among five Ugandan indigenous goat breeds and one commercial goat breed; constructed using a neighbour- joining tree from identity-by-state distances derived from 46 105 SNPs.

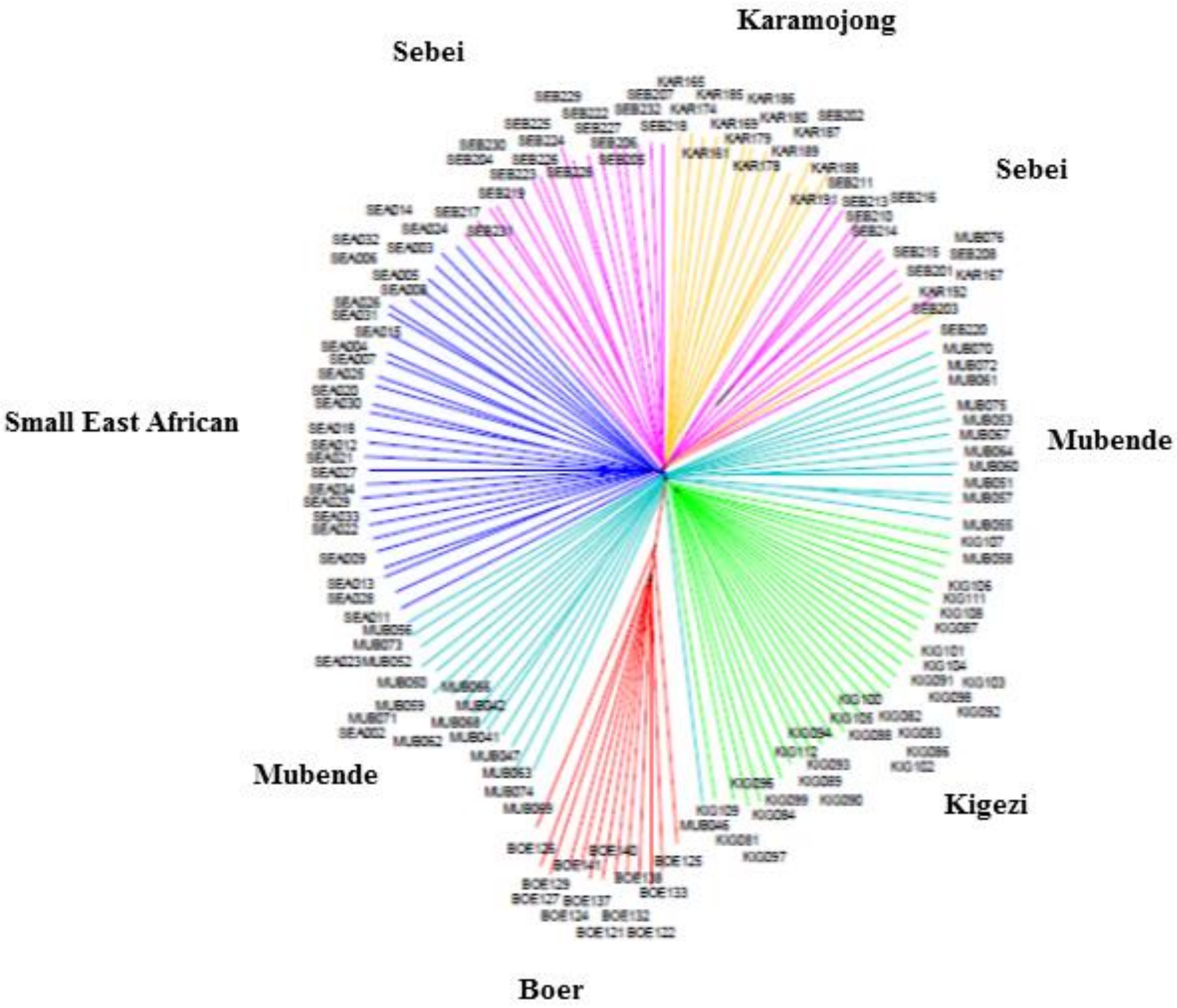

Supplement: Supplementary file 1 — Figure S1 Genetic relationships among five Ugandan indigenous goat breeds and one commercial goat breed; constructed using a neighbour‐ joining tree from identity‐by‐state (IBS) distances derived from 46 105 SNPs. [file AGE-49-59-s001.pdf]
