# Supplementary figures and images for: Genome‐wide population structure and admixture analysis reveals weak differentiation among Ugandan goat breeds
Source: Anim Genet. 2018 Jan 17;49(1):59–70. doi: 10.1111/age.12631 (PMC5838551; doi:10.1111/age.12631)

**Figure S2** Cross-validation error plot indicating the choice of the appropriate  $K$  value.

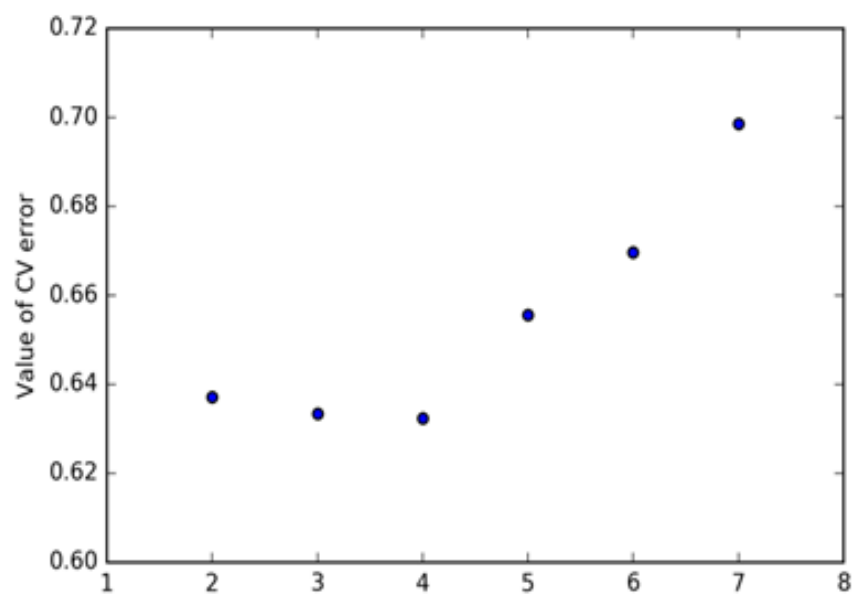

Supplement: Supplementary file 2 — Figure S2 Cross‐validation error plot indicating the choice of the appropriate K value. [file AGE-49-59-s002.pdf]
