# Supplementary material for: Genome‐wide population structure and admixture analysis reveals weak differentiation among Ugandan goat breeds
Source: Anim Genet. 2018 Jan 17;49(1):59–70. doi: 10.1111/age.12631 (PMC5838551; doi:10.1111/age.12631)
Supplement: Supplementary file 3 — Table S1 Significant f4 statistics for Ugandan goat breeds indicating gene flow in the breeds. [file AGE-49-59-s003.pdf]

Table S1: Results of four population tests showing significant f_4 statistics for Ugandan goats indicating gene-flow in the populations.

**Pop 1**	**Pop 2**	**Pop 3**	**Pop 4**	***f4 statistic***	**Std error**	**Z Score**
BOE	KIG	KAR	MUB	0.0050	0.0002	20.5959
BOE	MUB	KAR	KIG	0.0051	0.0003	20.4125
BOE	KIG	KAR	SEA	0.0027	0.0003	10.2101
BOE	KAR	KIG	SEB	0.0033	0.0003	12.5043
BOE	KIG	KAR	SEB	0.0022	0.0002	14.3987
BOE	MUB	KAR	SEA	0.0027	0.0003	10.8053
BOE	KAR	MUB	SEB	0.0034	0.0002	16.4090
BOE	MUB	KAR	SEB	0.0018	0.0002	12.1647
BOE	KAR	SEA	SEB	0.0020	0.0002	9.2824
BOE	SEA	KAR	SEB	0.0014	0.0002	8.2888
BOE	KAR	MUB	SEA	0.0014	0.0002	6.0396
BOE	SEA	KAR	MUB	0.0013	0.0002	5.6349
BOE	SEA	KAR	KIG	0.0014	0.0003	5.0805
BOE	SEB	MUB	SEA	0.0010	0.0002	4.4690
BOE	KAR	KIG	SEA	0.0013	0.0003	4.4078
BOE	SEB	KAR	KIG	-0.0011	0.0003	-4.0038
BOE	MUB	SEA	SEB	-0.0009	0.0002	-4.1880
BOE	SEB	KAR	MUB	-0.0016	0.0002	-6.4749
BOE	KIG	MUB	SEA	-0.0023	0.0002	-9.8169
BOE	MUB	KIG	SEA	-0.0024	0.0003	-9.4555
BOE	KIG	MUB	SEB	-0.0028	0.0002	-12.9529
BOE	MUB	KIG	SEB	-0.0033	0.0002	-14.3171
**Karamojong**						
KAR	SEB	MUB	SEA	-0.0004	0.0001	-4.5315
KAR	SEB	KIG	MUB	-0.0004	0.0001	-5.4680

Pop 1	Pop 2	Pop 3	Pop 4	*f4 statistic*	Std error (se)	Z Score
KAR	SEB	KIG	SEA	-0.0008	0.0001	-7.0525
KAR	KIG	MUB	SEA	-0.0037	0.0001	-29.0164
KAR	MUB	KIG	SEA	-0.0037	0.0001	-27.2518
KAR	KIG	MUB	SEB	-0.0062	0.0001	-47.4086
KAR	MUB	KIG	SEB	-0.0066	0.0001	-47.1151
KAR	KIG	SEA	SEB	-0.0025	0.0001	-18.3191
KAR	SEA	KIG	SEB	-0.0033	0.0001	-22.1784
KAR	MUB	SEA	SEB	-0.0029	0.0001	-23.6741
KAR	SEA	MUB	SEB	-0.0033	0.0001	-23.2563
**Kigezi**						
KIG	SEA	MUB	SEB	0.0029	0.0001	23.4857
KIG	SEB	MUB	SEA	0.0033	0.0001	28.0769
KIG	MUB	SEA	SEB	-0.0004	0.0001	-4.3254

BOE=Boer, KAR=Karamojong, KIG=Kigezi, MUB=Mubende, SEA=Small East African and SEB=Sebei goats. All Z-scores are significant ($p < 0.05$) indicating presence of gene-flow
