# Supplementary material for: Genome‐wide population structure and admixture analysis reveals weak differentiation among Ugandan goat breeds
Source: Anim Genet. 2018 Jan 17;49(1):59–70. doi: 10.1111/age.12631 (PMC5838551; doi:10.1111/age.12631)
Supplement: Supplementary file 4 — Table S2 Breed composition of cluster 1, 2, 3 and 4, interpreted as representative of Small East African, Karamojong/Sebei, Boer and Kigezi ancestries respectively, estimated for each individual belonging to goat populations from Uganda. [file AGE-49-59-s004.pdf]

Table S2 Breed composition of cluster 1, 2, 3 and 4, interpreted as representative of Small East African, Karamojong/Sebei, Boer and Kigezi ancestries respectively, estimated for each individual belonging to goat populations from Uganda.

Animal	Breed	Cluster 1 (SEA)	Cluster 2 (KAR/SEB)	Cluster 3 (BOE)	Cluster 4 (KIG)
BOE132	BOE	0.0000	0.0000	1.0000	0.0000
BOE133	BOE	0.0006	0.0676	0.9318	0.0000
BOE137	BOE	0.0000	0.0000	1.0000	0.0000
BOE138	BOE	0.0000	0.0000	1.0000	0.0000
BOE140	BOE	0.0000	0.0000	1.0000	0.0000
BOE141	BOE	0.0000	0.0000	1.0000	0.0000
BOE121	BOE	0.0000	0.0000	1.0000	0.0000
BOE122	BOE	0.0000	0.0000	1.0000	0.0000
BOE124	BOE	0.0000	0.0000	1.0000	0.0000
BOE125	BOE	0.0529	0.0179	0.7707	0.1586
BOE126	BOE	0.0426	0.0521	0.7572	0.1482
BOE127	BOE	0.0057	0.0723	0.9221	0.0000
BOE129	BOE	0.0028	0.0256	0.9715	0.0000
KAR161	KAR	0.0581	0.9079	0.0340	0.0000
KAR165	KAR	0.0941	0.8443	0.0465	0.0151
KAR167	KAR	0.2576	0.6264	0.0000	0.1159
KAR169	KAR	0.0450	0.8917	0.0575	0.0059
KAR174	KAR	0.0399	0.9026	0.0494	0.0081
KAR178	KAR	0.0000	0.9996	0.0004	0.0000
KAR179	KAR	0.0000	0.9833	0.0167	0.0000
KAR180	KAR	0.1140	0.7552	0.0167	0.1141
KAR185	KAR	0.0609	0.8804	0.0582	0.0005
KAR186	KAR	0.0564	0.8332	0.0465	0.0639
KAR187	KAR	0.0574	0.8779	0.0534	0.0112
KAR188	KAR	0.0495	0.8749	0.0756	0.0000
KAR189	KAR	0.1613	0.7839	0.0025	0.0522
KAR191	KAR	0.1011	0.8989	0.0000	0.0000
KAR192	KAR	0.2715	0.5331	0.0078	0.1876
KIG106	KIG	0.0666	0.0576	0.0000	0.8758
KIG108	KIG	0.0713	0.0000	0.0000	0.9286
KIG109	KIG	0.0661	0.2067	0.2001	0.5272
KIG107	KIG	0.0734	0.0377	0.1186	0.7702
KIG081	KIG	0.0608	0.1029	0.1766	0.6598
KIG088	KIG	0.0000	0.0000	0.0000	1.0000
KIG089	KIG	0.0000	0.0000	0.0250	0.9750
KIG090	KIG	0.0071	0.0000	0.0000	0.9929
KIG091	KIG	0.0000	0.0000	0.0000	1.0000
KIG092	KIG	0.0230	0.0000	0.1125	0.8645
KIG093	KIG	0.0000	0.0000	0.0000	1.0000

KIG094	KIG	0.0000	0.0000	0.0000	1.0000
KIG096	KIG	0.0613	0.0910	0.2393	0.6083
KIG097	KIG	0.0306	0.0679	0.1169	0.7846
KIG098	KIG	0.0000	0.0000	0.0000	1.0000
KIG099	KIG	0.0323	0.0408	0.0862	0.8407
KIG100	KIG	0.0000	0.0000	0.0000	1.0000
KIG101	KIG	0.0000	0.0000	0.0000	1.0000
KIG102	KIG	0.0000	0.0000	0.0000	1.0000
KIG103	KIG	0.0059	0.0238	0.1415	0.8288
KIG104	KIG	0.0000	0.0000	0.0000	1.0000
KIG105	KIG	0.0000	0.0000	0.0000	1.0000
KIG086	KIG	0.0000	0.0000	0.0000	1.0000
KIG087	KIG	0.0181	0.0000	0.0000	0.9819
KIG082	KIG	0.0000	0.0000	0.0000	1.0000
KIG083	KIG	0.0000	0.0000	0.0000	1.0000
KIG084	KIG	0.0553	0.0701	0.0351	0.8396
KIG111	KIG	0.0227	0.0517	0.0000	0.9256
KIG112	KIG	0.0548	0.0342	0.0557	0.8554
MUB051	MUB	0.2042	0.1914	0.0153	0.5891
MUB066	MUB	0.2156	0.1830	0.0529	0.5485
MUB067	MUB	0.2101	0.2692	0.0053	0.5154
MUB055	MUB	0.1947	0.2184	0.0183	0.5686
MUB061	MUB	0.1934	0.2385	0.0029	0.5652
MUB062	MUB	0.2301	0.1935	0.0624	0.5140
MUB068	MUB	0.2229	0.2407	0.0000	0.5364
MUB073	MUB	0.1814	0.3554	0.0149	0.4483
MUB074	MUB	0.1886	0.2921	0.1060	0.4133
MUB060	MUB	0.1979	0.1679	0.0989	0.5353
MUB053	MUB	0.2245	0.2429	0.0353	0.4973
MUB059	MUB	0.2020	0.2655	0.0274	0.5052
MUB052	MUB	0.2145	0.2636	0.0268	0.4951
MUB056	MUB	0.2468	0.2301	0.0192	0.5038
MUB064	MUB	0.2337	0.2147	0.0000	0.5516
MUB046	MUB	0.1721	0.1557	0.1850	0.4872
MUB047	MUB	0.2398	0.3022	0.0000	0.4580
MUB050	MUB	0.2129	0.2390	0.0060	0.5421
MUB057	MUB	0.1826	0.1349	0.1117	0.5708
MUB058	MUB	0.1902	0.1265	0.1078	0.5755
MUB070	MUB	0.1804	0.3547	0.0198	0.4451
MUB071	MUB	0.1879	0.4201	0.0000	0.3920
MUB072	MUB	0.2020	0.3417	0.0205	0.4357
MUB075	MUB	0.2241	0.2255	0.0230	0.5275
MUB076	MUB	0.2201	0.2717	0.0194	0.4888
MUB063	MUB	0.1852	0.2138	0.0935	0.5075

MUB069	MUB	0.1770	0.2619	0.1954	0.3657
MUB041	MUB	0.1666	0.2536	0.0635	0.5162
MUB042	MUB	0.1687	0.2501	0.1035	0.4778
SEA002	SEA	0.4465	0.3744	0.0121	0.1669
SEA003	SEA	0.5727	0.1914	0.0919	0.1440
SEA004	SEA	1.0000	0.0000	0.0000	0.0000
SEA005	SEA	0.6671	0.2003	0.0000	0.1326
SEA006	SEA	1.0000	0.0000	0.0000	0.0000
SEA007	SEA	1.0000	0.0000	0.0000	0.0000
SEA008	SEA	0.6615	0.1935	0.0168	0.1282
SEA009	SEA	0.6849	0.1517	0.0000	0.1634
SEA011	SEA	0.3394	0.3817	0.0000	0.2789
SEA012	SEA	0.7611	0.1270	0.0000	0.1118
SEA013	SEA	0.7908	0.1032	0.0000	0.1060
SEA014	SEA	0.7312	0.1663	0.0000	0.1025
SEA015	SEA	0.7088	0.1508	0.0038	0.1367
SEA018	SEA	0.6786	0.0999	0.0885	0.1330
SEA020	SEA	1.0000	0.0000	0.0000	0.0000
SEA021	SEA	0.8327	0.1116	0.0000	0.0557
SEA022	SEA	0.8111	0.1245	0.0000	0.0645
SEA023	SEA	0.7242	0.1586	0.0000	0.1172
SEA025	SEA	1.0000	0.0000	0.0000	0.0000
SEA026	SEA	1.0000	0.0000	0.0000	0.0000
SEA027	SEA	0.7184	0.1276	0.0154	0.1386
SEA028	SEA	0.8039	0.1081	0.0000	0.0880
SEA029	SEA	0.7834	0.1160	0.0282	0.0724
SEA030	SEA	1.0000	0.0000	0.0000	0.0000
SEA031	SEA	1.0000	0.0000	0.0000	0.0000
SEA032	SEA	0.7619	0.1319	0.0131	0.0932
SEA033	SEA	0.7943	0.0876	0.0000	0.1181
SEA034	SEA	0.8092	0.1026	0.0000	0.0882
SEA024	SEA	0.4307	0.3623	0.0000	0.2070
SEB232	SEB	0.1520	0.6956	0.0001	0.1523
SEB217	SEB	0.1825	0.5895	0.0373	0.1907
SEB218	SEB	0.1095	0.7059	0.0132	0.1714
SEB219	SEB	0.0220	0.8817	0.0000	0.0963
SEB207	SEB	0.1635	0.6530	0.0131	0.1703
SEB208	SEB	0.1980	0.6346	0.0012	0.1662
SEB228	SEB	0.1006	0.7817	0.0000	0.1178
SEB229	SEB	0.0585	0.8038	0.0320	0.1058
SEB220	SEB	0.0971	0.5467	0.0921	0.2641
SEB222	SEB	0.0779	0.7473	0.0100	0.1649
SEB223	SEB	0.0143	0.8756	0.0000	0.1101
SEB204	SEB	0.1373	0.6515	0.0114	0.1998

SEB230	SEB	0.0620	0.8328	0.0000	0.1052
SEB231	SEB	0.0275	0.9218	0.0000	0.0506
SEB203	SEB	0.1062	0.6934	0.0000	0.2003
SEB224	SEB	0.0245	0.8714	0.0403	0.0637
SEB225	SEB	0.0000	1.0000	0.0000	0.0000
SEB226	SEB	0.0000	0.9577	0.0000	0.0423
SEB227	SEB	0.0976	0.6782	0.0494	0.1749
SEB216	SEB	0.0403	0.8693	0.0020	0.0884
SEB214	SEB	0.0000	0.9485	0.0000	0.0515
SEB215	SEB	0.0271	0.8571	0.0000	0.1158
SEB201	SEB	0.1551	0.6791	0.0063	0.1594
SEB202	SEB	0.1196	0.6878	0.0238	0.1688
SEB205	SEB	0.0465	0.8083	0.0000	0.1452
SEB206	SEB	0.0645	0.7941	0.0000	0.1414
SEB210	SEB	0.0000	0.9473	0.0000	0.0527
SEB211	SEB	0.1247	0.7089	0.0146	0.1518
SEB213	SEB	0.0120	0.9199	0.0000	0.0682

BOE=Boer, KAR=Karamojong, KIG=Kigezi, MUB=Mubende, SEA=Small East African and SEB=Sebei goats.
